# Possibilities and limitations of advanced transmission electron microscopy for carbon-based nanomaterials

**DOI:** 10.3762/bjnano.6.158

**Published:** 2015-07-16

**Authors:** Xiaoxing Ke, Carla Bittencourt, Gustaaf Van Tendeloo

**Affiliations:** 1 EMAT, University of Antwerp, Groenenborgerlaan 171, 2020 Antwerp, Belgium; 2 Institute of Microstructure and Property of Advanced Materials, Beijing University of Technology, Beijing 100124, China; 3 Chemistry of Interaction Plasma Surface (ChiPS), University of Mons, Place du Parc 20, 7000 Mons, Belgium

**Keywords:** TEM, aberration-corrected, carbon, nanostructures, low-kV imaging

## Abstract

A major revolution for electron microscopy in the past decade is the introduction of aberration correction, which enables one to increase both the spatial resolution and the energy resolution to the optical limit. Aberration correction has contributed significantly to the imaging at low operating voltages. This is crucial for carbon-based nanomaterials which are sensitive to electron irradiation. The research of carbon nanomaterials and nanohybrids, in particular the fundamental understanding of defects and interfaces, can now be carried out in unprecedented detail by aberration-corrected transmission electron microscopy (AC-TEM). This review discusses new possibilities and limits of AC-TEM at low voltage, including the structural imaging at atomic resolution, in three dimensions and spectroscopic investigation of chemistry and bonding. In situ TEM of carbon-based nanomaterials is discussed and illustrated through recent reports with particular emphasis on the underlying physics of interactions between electrons and carbon atoms.

## Review

### 1 Introduction

For decades the electron microscopy community was strictly divided into biology on the one end and materials science on the other end. Meanwhile, however, the importance of “soft matter”, such as zeolites, porous materials, polymers, hybrid materials and carbon-based nanomaterials, is rapidly increasing. Optimal integration of soft matter materials into nanodevices calls for a fundamental interpretation of the structure of the materials, for which classical electron microscopy was actually poorly equipped. Soft matter materials are much more sensitive to electron beam damage compared to traditional metal or inorganic materials, and thus require imaging at low accelerating voltages. Working at lower voltages is not new; actually the first electron microscopes built by Ernst Ruska and Max Knoll were operated at low voltages [1]. However, because the resolution of an electron microscope scales with the wavelength of the electrons, the spatial resolution at low voltage was not sufficient for high resolution studies. Meanwhile, imaging at low voltage suffers dramatically from aberrations due to the imperfection of the electromagnetic lenses in the electron microscope. Driven by the pursuit of high resolution, manufacturers went on to higher operation voltages in the sixties and seventies of the twentieth century [2–5]. This came at the price of increasing beam damage, and consequently separated the study of hard matter (metal or robust inorganic materials) and soft matter.

Fortunately this has changed over the last decade. The introduction of spherical-aberration-corrected lenses [6] has paved the way out of this dilemma by improving the spatial resolution and increasing the signal to noise ratio at the same time. This has a dramatic impact when imaging at low accelerating voltage of 60–80 kV or even lower. At present the correction of spherical aberration (Cs correction) is a commonly used technique. Meanwhile, the correction of the chromatic aberration (Cc correction) in order to improve the uniformity of emitted electron beams has been demonstrated, but is still in an exploratory phase [7–9]. Particularly for relatively thick samples, the effect of a Cc corrector may be compromised by sample-induced chromatic effects, and the corrector is therefore only really useful to ultra-thin samples. An alternative is to use a monochromator to cut out tails in beam spreading, which reduces the intensity of the beam [10–12]. This again is of benefit for imaging soft matter materials because a lower electron dose means less damage to the material. More importantly, it improves the energy resolution for spectroscopic studies, which is another major step in the increase of performance of electron microscopes. Therefore, the advances of the instruments have led to a dramatic improvement in imaging at low voltage. Atomic resolution has been achieved at low voltages of 60–80 kV or even as low as 20 kV [13], and energy resolution has been increased up to 0.1 eV [12].

These recent progresses in electron microscopy offer an unprecedented opportunity to investigate beam-sensitive soft matter materials, in particular carbon-based nanomaterials, while only doing little damage to the samples. Carbon is one of the most essential elements on earth, named after the Latin word of “carbo” referring to charcoal. The use of charcoal, soot and coal dates back to prehistoric times, when nano-structured carbon materials already existed. Analysis of prehistoric cave paintings in Altamira (Spain) and Lascaux (France) has revealed the presence of carbon nanoparticles [14–16]. Carbon nanoparticles were also essential ingredients in inks and printing pastes used over centuries in various cultures [17]. Another example are carbon nanotubes (CNTs) [18] which found their way into the secret recipe of ultra-sharp Damascus steel, which dates back to seventeenth century, and are believed to be responsible for its extraordinary mechanical properties [19–21]. In the materials mentioned above, nano-structured carbon was used as an essential part to tailor their properties and characteristics. Carbon nanoparticles are mixed with collagen-derived animal glue to achieve a high homogeneity when dispersed into a colloidal solution. CNTs in Damascus steel are found to encapsulate cementite nanowires which might account for its super-plastic behavior. More recently, the report of graphene [22] has triggered extensive studies on its rich physics and has opened up wide applications in photoelectric devices, catalysis supports, battery electrodes, and many more.

The research of carbon nanohybrid materials, including both the fundamental study of carbon nanostructures and the understanding of interface formation between nano-carbon and the host matrix, is essential to the understanding of their unusual electronic, mechanical or thermal behavior, and further assists the optimal design of carbon-based nanodevices in a smart and sustainable manner. Such structural and chemical characterizations become possible at both high spatial resolution and high energy resolution with only limited beam damage thanks to major advances in TEM [23]. In addition to static imaging of the carbon nanostructure, the controlled interaction between electrons and carbon atoms may add a new dimension to the imaging, where electrons probe the carbon lattice during imaging and therefore lead to a dynamic investigation based on the fundamental physics of the materials.

In this review, we start from a brief outline of electron microscopy improved by aberration correction at low voltage, with an emphasis on the interaction between electrons and carbon atoms. The different applications of electron microscopy for carbon-based nanomaterials are then reviewed, including structural imaging at atomic resolution and in three-dimensional (3D) reconstruction, spectroscopy of the chemistry at defects and interfaces, and in situ TEM under external stimuli along with dynamic TEM.

### 2 Basics of TEM: Lower the voltage

#### 2.1 Aberration correction

Electron beams accelerated at high voltages (tens to hundreds of kilovolts) have wavelengths far below the scale of inter-atomic distances of all materials, so in principle they are able to resolve the structures. As a general rule, the resolving power scales with the wavelength of the accelerated electrons. Using de Broglie equations, the wavelength of a 200 kV electron beam is approximately 0.025 Å, whereas that of 80 kV and 60 kV is about 0.04 Å and 0.05 Å, respectively. It can be seen that even electron beams accelerated by the low voltages of 80 kV and 60 kV are sufficient in resolving the inter-atomic distances at sub-angstrom level. However in conventional TEM, the resolution at 200 kV is usually approx. 1–2 Å and it is even lower for 120 kV or 80 kV. This raises the question: What limits the resolution of TEM to such a large degree and how to overcome it?

**Figure 1:**
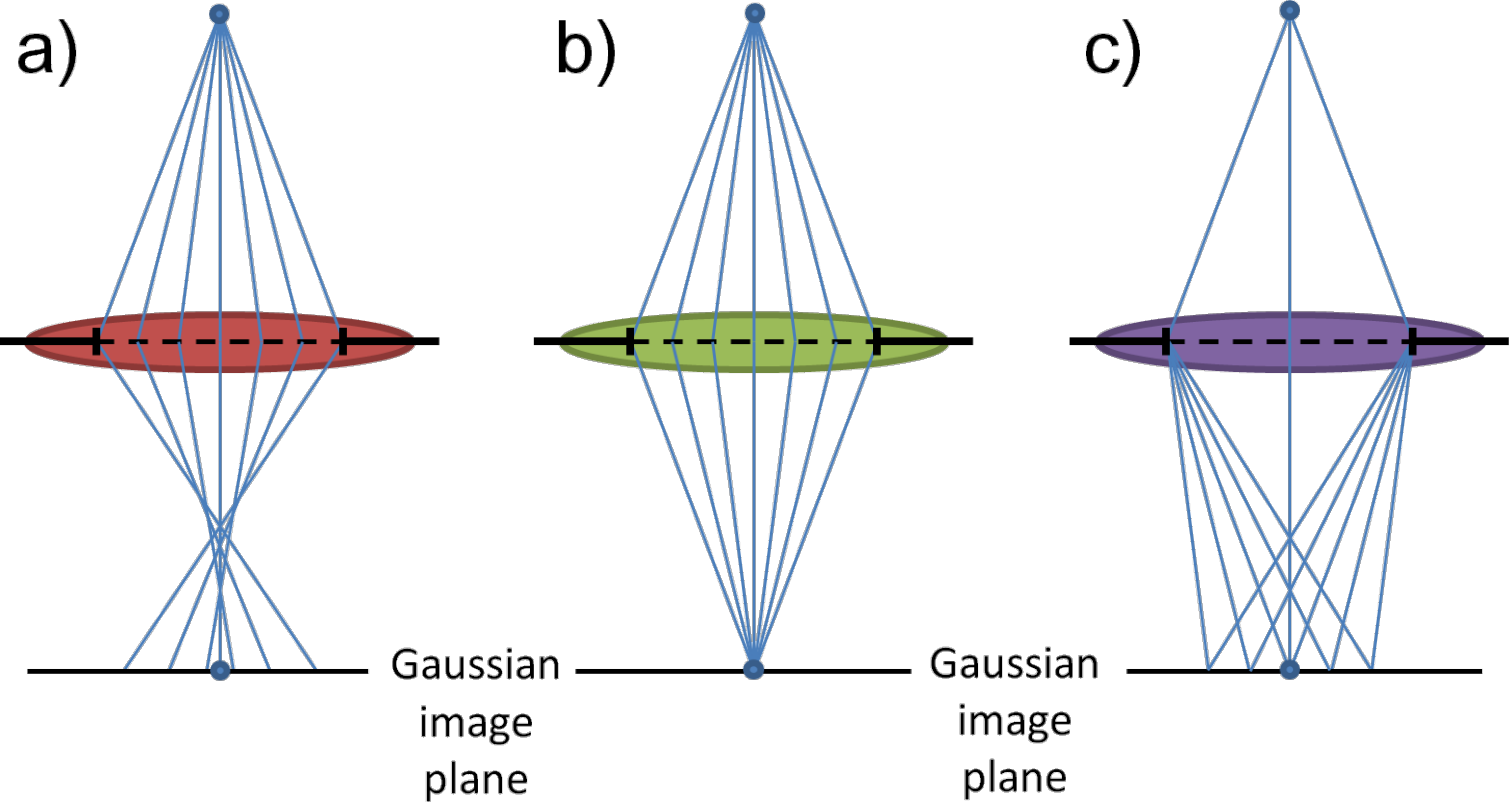
Illustration of (a) spherical aberration (b) ideal lens and (c) chromatic aberration.

To answer this question we need to consider that the formation of an image relies not only on the illumination source, but is also influenced by a series of electromagnetic lenses before and after electrons interacting with the specimen. Generally speaking, imperfections in the optical system introduce lens aberrations, which are continuously magnified during the propagation of the electron waves. Spherical aberrations and chromatic aberrations are the most well-known aberrations to lower the resolving power of an electron microscope [24]. Spherical aberration induces a blurring of the image in the focal plane, because light rays through the center of the lens and through the edge of the lens deviate when intersecting with the optical axis. Chromatic aberration on the other hand, induces blurring of the image because light rays of different wavelengths (due to the energy spread) fail to intersect with each other on the optical axis. Electron waves of shorter wavelengths and higher energies are refracted stronger. Spherical aberration and chromatic aberration are schematically shown in Figure 1.

It can be seen that lens aberrations limit the resolving power of electron microscopes. For simplicity, the point resolution of a HRTEM is expressed by Equation 1 [25]. A quantitative description of different lens aberrations in electron microscopy has been extensively discussed in the literature [24].

[1]
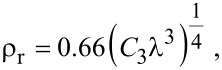


where λ is the wavelength of the electrons and *C*_3_ is the third-order spherical aberration coefficient of the objective lens. It must be noted that *C*_3_ has a dominant influence on imaging; higher order aberrations are neglected here for simplicity.

**Figure 2:**
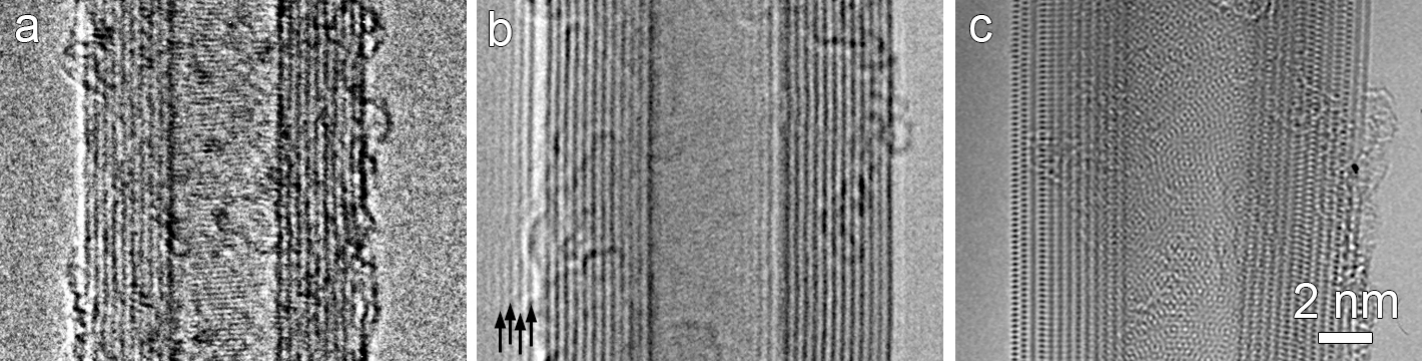
HRTEM images of MWCNTs acquired (a) at 200 kV without Cs correction; (b) at 120 kV without Cs correction, where delocalization is indicated by arrows; and (c) at 80 kV with Cs correction using a monochromated beam.

The difference in the point resolution is therefore dramatically determined by the value of *C*_3_. A conventional TEM has a *C*_3_ value in the range of millimeters, which results in a reduction of the resolving power. Using a multi-walled carbon nanotube (MWCNT) for demonstration, a high resolution TEM (HRTEM) image acquired at 200 kV using a conventional FEI Tecnai G^2^ microscope is shown in Figure 2a, where the spatial resolution is about 1.5 Å. When the accelerating voltage is lowered to 120 kV, not only the resolution is reduced, but there is also a strong contrast delocalization, which relates to spherical aberration [6,26]. A typical example of delocalization is shown in Figure 2b, where a MWCNT is imaged at an operating voltage of 120 kV using the same Tecnai G^2^ microscope. A strong delocalization projected in the vacuum in the vicinity of CNT walls is indicated by arrows.

The increased delocalization and limited resolution complicates the interpretation of nanostructures when studied at low voltages. The most straightforward solution, as suggested by Equation 1, is to decrease the value of *C*_3_ by reducing the lens aberration. However, although this suggestion was known for a long time, it was not implemented in the beginning of electron microscopy. The first Cs correctors were developed in the 1990s [6]. State-of-art aberration corrected TEMs (AC-TEM) are now commercially available and the number of worldwide users is increasing exponentially. The development of aberration correctors are not discussed here, but an impressive result using AC-TEM to image MWCNTs at a voltage of 80 kV is demonstrated in Figure 2c to be compared with Figure 2a–b. Neighboring atom columns on the graphitic shell can be unambiguously distinguished. The delocalization is suppressed to a large degree, providing a straightforward interpretation of the surface and interface structure.

After the successful implementation of Cs correctors, there have been strong efforts to also correct chromatic aberration, however these corrections turn out to be more delicate and are still to be improved. Chromatic aberration becomes more important at lower voltages due to a wider relative energy spread compared to high voltage electron waves. An alternative to reduce chromatic aberration is often implemented by cutting electron beam tails using a monochromator, where the energy spread is considerably narrowed. The highly-aligned beam energy minimizes the effect of chromatic aberration, and hence improves the information limit to the sub-angstrom level at low operating voltages [27]. Actually, Figure 2c was obtained using a monochromated electron beam of 80 kV together with Cs correction. It must be noticed that the use of a monochromator will reduce the beam intensity due to a partial removal of the energy spectrum, which effectively minimizes possible damage to the structure, as will be discussed in the next subsection.

The previous discussion of Cs correctors applies to the post-field of the objective lens, which corrects the electron trajectory of the exit electron wave after interaction with the specimen, and provides a more straightforward interpretation of the projected potential of the specimen. When the correction is used in bright-field imaging using a parallel beam the corrector is referred to as “image corrector”. When the Cs corrector is applied to the electron beam before interacting with the specimen, and forms a highly converged electron probe, it is referred to as “probe corrector”. In the latter case we are talking about scanning transmission electron microscope (STEM), where an electron probe scans over a desired area to obtain local structural or spectroscopic information. When working with a highly converged beam at low voltage, a monochromator, reducing the beam energy spread, is desired in combination with a probe corrector.

Introduction of AC-TEM has provided unprecedented spatial resolution at low voltages and has thus largely benefited the studies of carbon-based nanomaterials and nanohybrids. Imaging conditions under 80 kV [28], 60 kV [29], even 30 kV [30] and 20 kV [13] have been demonstrated in studying carbon-based nanostructures.

#### 2.2 Interaction between the electron beam and carbon-based materials

Electron microscopes use electron waves to resolve atomic structures and overcome the diffraction limit of optical microscopes. However, different from optical microscopes, accelerated electrons carry high energy and interact with the material in a highly dynamic manner. Elastically scattered electrons, inelastically scattered electrons (for electron energy loss spectroscopy, i.e., EELS) and X-rays (for energy dispersive X-ray spectroscopy, i.e., EDX), all uniquely characterize the studied materials. Chemical compositions, electron fine structures, even the phonon vibrations [31] produced by electron–matter interactions can be acquired, which is quite exciting for a detailed study. Therefore, electron beams are more than an illumination source, and can be considered as a tool in probing the intrinsic physics and chemistry of the investigated materials.

One should be concerned however, that such probing may modify the structure and introduce artefacts. In this context, low voltage is critical, particularly for beam-sensitive carbon-based nanostructures. The most well-known artefact to be avoided in carbon-based nanomaterials is the so-called “knock-on damage”. When high energy electrons collide with the carbon lattice and the momentum transferred during the collision exceeds the binding energy of the carbon–carbon bonds, carbon atoms can be displaced in competition with a spontaneous recombination, resulting in defects in the lattice, i.e., knock-on damage.

If we consider the collision only, the maximum kinetic energy transferred from the accelerated electrons to the atoms during the collision, which is regarded as a pure elastic head-on one, is expressed as follows [32]:

[2]
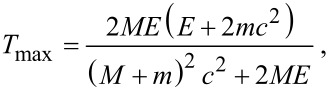


where *M* and *m* are the masses of the atom and electron respectively, *c* is the speed of light, and *E* is the energy of the electron. We have calculated *T*_max_ for different accelerating voltages: The results are approx. 25 eV, approx. 20.5 eV, approx. 16 eV and approx. 11.8 eV for incident electrons of 120 kV, 100 kV, 80 kV and 60 kV respectively.

**Figure 3:**
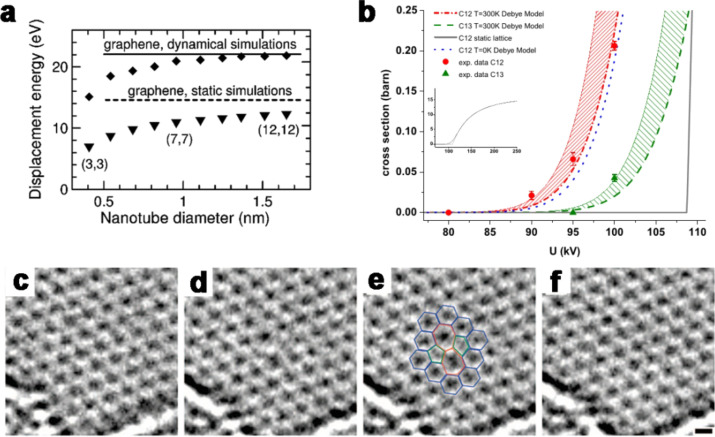
(a) Threshold energy *T*_d_ needed to displace carbon atoms from armchair multi-walled carbon nanotubes (SWCNTs) and graphene calculated dynamically (diamonds) and statically (triangles) as a function of tube diameter; the lines are the corresponding results for graphene; reproduced with permission from [33], Copyright (2005) American Physical Society. (b) Measured and calculated knock-on displacement cross sections. The lower boundary of the shaded areas corresponds to the calculated cross section, while the upper boundary is twice the calculated value. The inset shows the calculations for ^12^C, 300 K and static lattice on a larger energy range; reproduced from [35], Copyright (2012) American Physical Society. (c–f) Metastable Stone–Wales (SW) defects found in HRTEM image sequence: (c) unperturbed lattice before appearance of the defect, (d) SW defect, (e) same image with atomic configuration superimposed, (f) relaxation to unperturbed lattice (after ca. 4s); reproduced with permission from [37], Copyright (2008) American Chemical Society.

**Figure 4:**
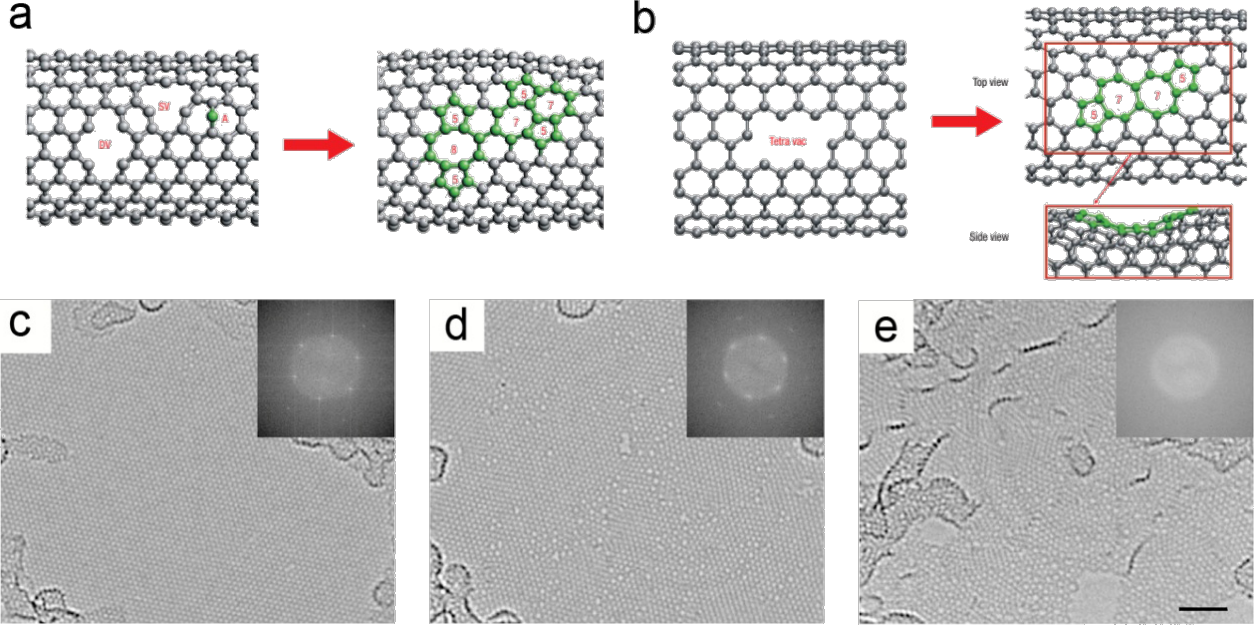
(a–b) Reconstruction of the atomic network of a CNT near vacancies and adatoms is predicted by atomistic computer simulations. SV, DV, tetravacancies and adatoms may transform into a Stone–Wales (5–7–7–5) defect and 5-8-5 defects; reproduced with permission from [43], Copyright (2007) Nature Publishing Group. (c–e) Amorphization of graphene demonstrated by three TEM images and their corresponding Fourier transformations (insets) at (c) a low irradiation dose (1.25 × 10^8^ e^−^/nm^2^), (d) an intermediate dose (2.94 × 10^9^ e^−^/nm^2^) and (e) a high dose (9.36 × 10^9^ e^−^/nm^2^); reproduced with permission from [36], Copyright (2014) Nature Publishing Group.

The threshold energy, *T*_d_, needed to displace a carbon atom has been calculated for defect-free graphene as an ideal case. Earlier results have reported a *T*_d_ of 15 eV and 22 eV calculated statically and dynamically, as shown in Figure 3a [33]. More recent studies using molecular dynamics simulations based on tight-binding density functional theory [32] and first principle calculations [34] have agreed on a *T*_d_ of 23 eV and 22 eV, respectively, corresponding to an accelerating voltage of about 110 kV using Equation 2. However, HRTEM on defect-free graphene at 100 kV causes damage to the sample, including pentagons, heptagons and octagons [35]. The experimental results clearly show that a *T*_d_ of 23 eV and 22 eV (corresponding to an incident beam of approx. 110 kV) is overestimated. Such a difference may indicate that the irradiation of graphene with electrons includes more complicated interactions than only direct knock-on collisions with the carbon nuclei. A better fit is found by taking lattice vibration into account [35], rather than a static lattice approximation used in earlier literature. The damage cross-section rises is nearly zero at 80 keV and increases monotonically afterwards, as shown in Figure 3b. The result is well supported by the experimental results of extensive defect formation at 100 keV [36] (see below in Figure 4c–e) and occasional defects observed at 80 keV as shown in Figure 3c–f [37].

On the other hand, a lower threshold is expected for graphene with defects [38]. A typical example is the edge of a graphene sheet where an electron energy below approx. 50 keV is recommended to minimize the knock-on damage [39], and even this already low value is suggested to be still overestimated. Meyer et al. commented that extended holes (rather than a knock-on vacancy) grow over a wide range from 20 to 100 keV, or even below 20 keV. A mechanism of beam-induced etching with residual water or oxygen in the system is therefore suggested [40]. The strong anisotropic tubular structure of CNTs leads to an anisotropy of the atomic displacement threshold [32,41–42]. The scattering geometry naturally contributes to the variation of the knock-on threshold depending on the nanotube diameter [33]. As shown in Figure 3a, CNTs invariably have a lower knock-on threshold than graphene, whereas the CNTs of smaller diameters are more sensitive to knock-on damage.

The message concluded from the literature study is that the knock-on threshold for defect-free graphene can be regarded to be 80 keV, although the dynamics of the carbon atoms cannot be entirely excluded. A lower voltage of 60 keV is generally safe for analyzing most carbon-based nanostructures. Great care must be taken in investigating extensive defects such as a graphene edge, where a lower voltage may not necessarily mean the better solution, as beam-induced etching will occur [40].

Following the knock-out of carbon atoms, vacancy formation and the subsequent reconstruction of large even-number vacancies takes place, as discussed in detail in [43]. One example is shown in Figure 4a,b. When carbon atoms in CNTs are displaced by electron irradiation, single vacancies (SV), divacancies (DV), and even tetravacancies can be created; a schematic illustration is presented in Figure 4a,b. Displaced carbon atoms may form adatoms (A) on the lattice. Atomistic computer simulations predict that the reconstruction of the atomic network near vacancies and adatoms is very likely to happen, resulting in an agglomeration of 5- to 8-membered rings [44–45]. As shown in Figure 4a, a SV and an adatom may form a metastable Stone–Wales (5–7–7–5) defect or a 5–8–5 defect. A tetravacancy could transform into a Stone–Wales defect, too (Figure 4b). As discussed above, electron beams of voltages exceeding 80 kV have a larger probability in displacing carbon atoms, resulting in structural disorder. Severe continuous illumination may eventually lead to complete amorphization of the lattice at room temperature [36]. As demonstrated in Figure 4c–e, an ordered graphene lattice is transformed into a disordered two-dimensional (2D) carbon glass by continuous irradiation at 100 keV.

It has been suggested to study carbon-based nanostructures at low voltage in order to suppress knock-on damage (elastic collision), allowing for a damage-free study in both TEM and STEM. One may point out that a decrease of the accelerating voltage has the disadvantage of increasing the damage cross section introduced by inelastic scattering [46]. This may lead to effects such as ionization damage and sample heating. These are indeed major concerns in studying polymers and biomaterials [23]. Fortunately, carbon-based nanomaterials, such as CNTs or graphene-based nanohybrids, have excellent electric and thermal conductivity [47–48] and suffer only slightly from damage related to inelastic scattering. Although the previous discussion has pointed out that low operating voltages increase the damage of large holes possibly due to etching, imaging conditions of 60 keV and 80 keV are generally accepted as reasonable. Additionally, the increase of the scattering cross section at low voltage improves the signal to noise ratio for light elements as carbon and results in an enhancement in contrast [38,49].

Following the discussion on lowering the voltage, the dose of incident electrons must be considered as well. Electrons accelerated by voltages close to the knock-on threshold of 80 kV or 60 kV may still displace carbon atoms in a graphene lattice, particularly the ill-bonded atoms at defects, surfaces or interfaces. Lowering the dose can therefore reduce such damage. This can be achieved by, e.g., spreading the electron beam, increasing the spot size, or minimizing the exposure time. In addition, the condenser aperture or spotsize can be tuned to reduce the beam intensity. The sensitivity of the CCD camera then becomes critical in order to maintain an acceptable noise-to-signal ratio. A recent development in an advanced high-resolution fast-detection camera (K2-IS camera from Gatan Inc.) has made a significant improvement in both sensitivity and resolution by the elimination of the traditional scintillation process and the capture of electrons directly on a CMOS (complimentary metal-oxide semiconductor) sensor up to 1600 fps [50]. The high sensitivity and fast acquisition in detecting has made possible the automated and ultra-fast acquisition of a series of under-exposed images from the same region. After drift correction, the images of such a sequence are stacked and can thus provide a HRTEM image with an acceptable signal to noise ratio with only limited damage to the sample.

The introduction of a high-speed detector may also have an impact on increasing the time resolution. In a molecular dynamics simulations on the reconstruction of vacancies, the time scale is often restricted to picoseconds [45], whereas in TEM the time resolution is at the order of 1 s [51] or 80 ms [52]. Under these conditions, it is more likely that the resulting image shows the time-relaxed state of the sample. A high-speed detector may facilitate the imaging of more intermediate states of the carbon dynamics. To push the time resolution to the limit, a revolutionary change in the electron source technology is required. Progress is being made by groups at Caltech (http://www.ust.caltech.edu/press/uem1.html) and Lawrence Livermore National Laboratory (https://www-pls.llnl.gov/?url=science_and_technology-materials-dtem). They reported TEM results with temporal resolutions of nanoseconds and picoseconds and spatial resolutions of angstroms and nanometers are reported [53–56]. Nevertheless, few results are reported at voltages below 80 kV.

#### 2.3 Imaging conditions and image interpretation

An important question to be answered is: How to determine the ideal imaging conditions? Although AC-TEM, which allows for atomic resolution at low operating voltages, is being promoted to be the standard for research facilities, should we abandon imaging at under more conventional conditions, e.g., 200 kV without Cs correctors? Dating back to the early nineties of last century when CNTs were reported first, much effort was devoted to resolve the structures of CNTs using TEM without aberration correction. Although the exact structure of CNTs could not be directly imaged by HRTEM because of the limited spatial resolution, attention was focused on unfolding the mystery through electron diffraction. Different from the phase contrast projected in the imaging plane, information projected in reciprocal space is much less influenced by lens imperfections. In addition, diffraction patterns reflect the kinetic and dynamic scattering of electrons when interacting with the unique structure of CNTs. Fundamental understanding of the physics during the interaction between the electrons and the carbon lattice is crucial. Together with the real space imaging at higher voltages, the nanostructure and the chirality of CNTs was successfully resolved [57–59].

It is an example to be well remembered. Firstly, the fundamental physics of the lens optics as well as that of the electron–matter interaction must be taken into account when it comes into the interpretation of TEM images. Secondly, a proper understanding of the lens optics in electron microscopes opens up more possibilities in choosing the most appropriate imaging condition for studies of different purposes. Not all carbon-based nanomaterials require imaging using monochromated AC-TEM at 80 kV. When the irradiation damage can be reasonably controlled by limited exposure time, high voltage with a low dose can be considered as an alternative. Thirdly, the development in AC-TEM offers unprecedented spatial resolution to be achieved at low voltage, which (1) provides a relatively straightforward (S)TEM image readily to be interpreted; (2) allows for an extensive timescale for structural investigation before breaking down. Consequently, local structures such as defects or active sites can be investigated in detail. More interestingly, the interaction between electrons and the materials can be imaged in a dynamic manner. Evolution of the structural defects, for instance, is evoked by electrons as a tool and imaged simultaneously. From this point of view, the electron beam is more than an illumination source in producing projections of the investigated materials as in a “shadow play”. It is, to be more accurate, the process or the result of an electron–matter interaction that is projected. This fact is the key to interpreting TEM images or TEM-acquired spectra.

### 3 Applications to carbon-based nanomaterials

#### 3.1 Structural Imaging: (atomic resolution and 3D)

Earlier studies using TEM to image the structures of carbon-based nanomaterials have limited spatial resolution, as shown above in the example of CNTs. Through the developments in AC-TEM nowadays, not only atomic resolution can be achieved, but also the obtained phase contrast suffers much less from the lens aberration, providing easily interpretable images. Local defects such as vacancies, dislocations, grain boundaries and strain can be revealed in great detail. The fundamental understanding of CNTs, particularly of graphene in the past decade, have largely benefited from the development in electron microscopy.

**Figure 5:**
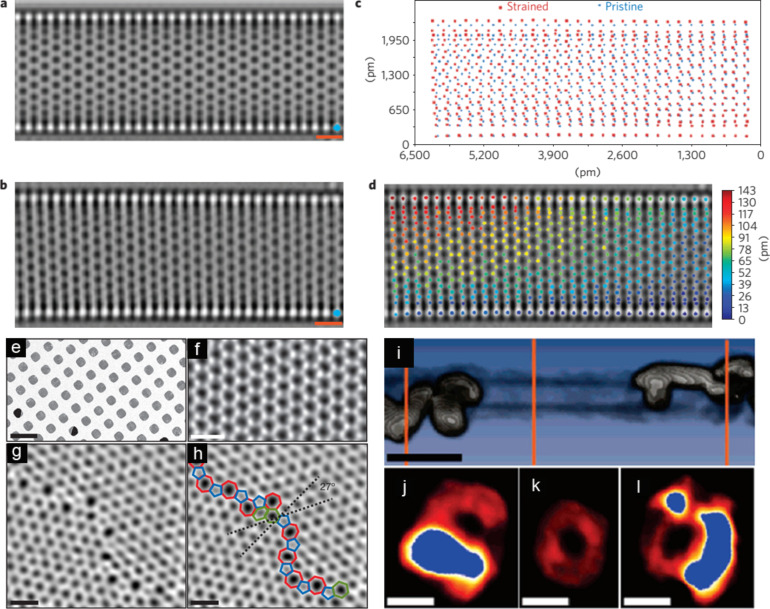
(a–d) Obtaining a 2D displacement map of a (28,0) SWCNT; reproduced with permission from [60], Copyright (2011) Nature Publishing Group. (a) HRTEM image simulation of a (28,0) SWCNT. Scale bar: 500 pm. (b) Experimental AC-TEM image of a (28,0) SWCNT with strain. Scale bar: 500 pm. (c) Overlay of atomic column positions measured from a (28,0) SWCNT in simulated (a) (blue) and experimental (b) (red) HRTEM images. (d) 2D displacement map, overlaid on top of the HRTEM form (b). (e–h) ADF-STEM images of graphene crystals; reproduced from [62], Copyright (2011) Nature Publishing Group. (e) Scanning electron micrograph of graphene transferred onto a TEM grid. (f) ADF-STEM image showing the defect-free hexagonal lattice inside a graphene grain. (g) Two grains intersect with a relative rotation of 27°. An aperiodic line of defects stitches the two grains together. (h) The image from (g) with the pentagons (blue), heptagons (red) and distorted hexagons (green) of the grain boundary outlined. Panels f–h were low-pass-filtered to remove noise; scale bars: 5 Å. (i–l) 3D reconstruction of a CNT in contact with Pd islands; reproduced with permissionfrom [83], Copyright (2007) American Chemical Society. (i) Volume rendering of the geometrically reconstructed CNT (black) with iso-intensity surface of Pd islands (white). Scale bar: 10 nm. (j-l) Cross-section views of the orange slices in (i). Scale bars: 4 nm.

Taking CNTs again as an example, the direct imaging of a zig-zag single-walled CNT (SWCNT) at atomic resolution using AC-TEM operated at 80 kV is able to reveal atomic displacements with picometer precision [60], as demonstrated in Figure 5a–d. The CNT is determined to have a chirality of (28,0) as shown in Figure 5b. By comparing to a simulated CNT with the same chirality (Figure 5a), a displacement map can be obtained (Figure 5c–d) at picometer precision, which further reveals the strain distribution. Strain induced by bending can be mapped in two dimensions, and further proposed to be a dominant non-uniform shear strain. The strain in the nanotube is associated to the modification of its intrinsic physical properties, including bandgap variation and quantum transport disturbance. Therefore, a detailed study of how the atomic structure responds to strain has contributed to the fundamental understanding of the physics of CNTs and other related nanostructures.

Similarly, AC-TEM has contributed significantly to the fundamental understanding of graphene. Its superior physical properties derived from its unique 2D nanostructure triggered extensive research about how defects in graphene can influence its properties and can further be utilized to tailor its macroscale behavior. By directly imaging a single layer of graphene at atomic resolution, the role of vacancies and ad-atoms can be studied in detail [28]. Non-hexagonal lattices in which C–C bonds are no longer sp^2^-hybridized have been identified. These included pentagons, heptagons, and octagons, which are usually paired to form 5–7 pairs or even 5–8 pairs [28,43] as demonstrated in Figure 4a,b. The imaging with AC-TEM confirms the presence of these defects in graphene, and provides evidence for the variations in its electronic properties, mechanical properties and thermal properties [61]. An example is shown in Figure 5e–h, where a grain boundary is imaged on a single graphene layer using annular dark field scanning transmission electron microscopy (ADF-STEM) acquired at 60 kV. An extensive arrangement of the 5–7 pairs together with distorted hexagons is revealed (Figure 5g,h) [62]. It can be deduced that electronic scattering and phonon scattering are very likely to be disturbed at the boundary. The fundamental studies on the atomic structure of graphene have a significant impact on the large-scale applications of graphene. Graphene synthesized through chemical vapor deposition (CVD) often exhibits a polycrystalline morphology with defects such as grain boundaries [62–63] and therefore suffers from a degradation of its physical properties. Characterization of the intrinsic defects and their further relation to the synthesis conditions, e.g., substrate lattice mismatch and annealing temperature is therefore of great importance.

In addition to the fundamental research on the structures of carbon-based nanomaterials, advanced electron microscopy has provided great opportunities to investigate functionalized carbon-based nanomaterials as well. Carbon nanohybrids found widespread use in nanoscience and nanotechnology: as building blocks for nanoelectronics [64], for drug delivery in biomedicine [65], and as bio-imaging agents when decorated with magnetic nanoparticles [66], just to name a few. The investigation of the interfaces of nanohybrids is often crucial in optimizing their design toward eventual applications. Two important examples are the studies of the interface formation at electrical contacts between CNTs and a metal, which needs to be understood for applications in nanoelectronic devices [67], and nanocatalysts where CNTs are used as conducting bed for efficient charge transfer [68–69]. Systematic studies of different metals on CNTs has been reported, including Au, Pt, Pd, Rh, Cu and Ti [67,70–75]. Early studies using HRTEM have revealed that Au and Pt form mostly well-crystallized nanocrystal islands with limited contact areas with the CNTs walls, whereas Pd and Rh form triangular shaped nanoparticles on CNT walls with an increased area at the contact interface, and Ti forms an amorphous film with continuous coverage around the CNTs. These results are further associated with the wettability of different metals on the CNT surface and explained by electron affinity and binding energy through DFT calculations [67]. Similarly, the electron affinity and binding energy difference can influence the reactions inside the CNTs, although the interior of the CNTs is regarded as inert due to its concave surface [76–77]. Recent studies using AC-TEM at the atomic scale have revealed that transition metals, such as W, Re and Os, encapsulated inside CNTs, can react with inner wall graphitic layers, depending on their affinity and bonding energy with the graphitic layers [78], or even stimulate the formation of nanometer-sized protrusions [77].

Detailed TEM studies on the metal/CNTs interface have also noticed a certain degree of bending in graphitic layers [79]. Conventional 2D imaging is insufficient in this case, as it acquires a projection of a 3D object, resulting in misinterpretation due to the lack of information along the projected direction. A better solution is to use 3D electron tomography to reveal the overall deformation. By acquiring a series of projections over a tilt range, the 3D structure can be reconstructed. As one of the most developed new TEM methods in the past ten years, 3D electron tomography has attracted tremendous attention since the first review paper on 3D electron tomography published by P. Midgley in 2003 [80]. Detailed discussions on data acquisition [80–81] and the reconstruction algorithm [82] can be found elsewhere. The result of the 3D reconstruction of a Pd–CNT interface is in parts presented in Figure 5i–l [83]. It has been reported that the deformation of Au-contacted CNT walls is more prominent compared to Pd-contacted CNT walls (not shwon), which could be associated to higher wettability of Pd over Au nanoparticles on one hand. On the other hand, a deformation mechanism through elastic strain relaxation is also proposed which attributes the deformation to lattice mismatch [79]. Whichever the driving force is, the geometric distortions in the graphitic lattice have been clearly evidenced by 3D TEM, indicating a significant difference in the electronic structure at the metal–CNT contact. The consequent resistance change at the contact is believed to contribute to the already present Schottky barrier in metal–CNT contacts, providing an alternative perspective in studying metal–CNTs contacts.

Undoubtedly, advances in TEM have offered unrivaled opportunities in studying carbon nanostructures in both 2D and 3D in a straightforward manner. Following the large improvement in the spatial resolution of 2D imaging, atomically resolved 3D reconstruction has been achieved and demonstrated on Au nanorods [84]. Encouragingly, only a few projections are required for the reconstruction thanks to an improvement of the reconstruction algorithm [82,84]. For beam-sensitive materials, such as carbon-based nanomaterials, a limited exposure time is preferred. Therefore this novel reconstruction method with a few projections can be extremely useful in obtaining the 3D structure of carbon-based materials at higher spatial resolution and moving it from the nanometer scale [85] toward the atomic scale.

#### 3.2 Advanced spectroscopy of carbon-based materials

In contrast to structural imaging which uses elastically scattered electrons, chemical and electronic structure information can be obtained simultaneously using inelastically scattered electrons or X-rays emitted during electron–matter interaction. By combining analytical techniques including EELS and EDX, modern electron microscopy reaches its ultimate potential in both higher spatial resolution and higher energy resolution.

Compared to EDX which is typically used to detect heavy elements, EELS is more frequently used for light elements and therefore carbon-based nanostructures. Generally speaking, the inelastic scattering of the incident electrons, either with the tightly bound inner shell electrons or with more loosely bound valence electrons, can cause atomic electrons to be excited to unoccupied states, and is reflected as a loss in energy when recorded using an EELS spectrometer. Not only elements can be identified using EELS, but the fine structure of the spectral profiles also reflects the specific electronic structures and chemical bonds [86–87]. Detailed analysis of the low-loss or valence region of an EELS spectrum (<50 eV) allows one to study the band structure and in particular the dielectric function of a material. In addition to the collective electron excitation modes marked by characteristic plasmon peaks, the joint density of states above the Fermi level is encompassed within the valence-loss region of an EELS spectrum. In combination with theoretical calculations and simulations detailed local information can be obtained. EELS is close to near-edge X-ray absorption fine structure (NEXAFS) analysis used to probe electronic states [88]. One major difference is that the spatial resolution in TEM is much higher than that of in NEXAFS. The bottleneck of EELS was the energy resolution, because in classical filaments or in a “warm emission gun” (Schottky filament) the energy spread of the emitted electron beam is fairly broad and the resolution of the EELS was of the order of 1 eV. The introduction of a chromatic aberration corrector or alternatively a monochromator allows one to overcome this shortage. By reducing the energy spread as discussed in Section 2, the energy resolution can now be down to the 100 meV range and even below. Replacement of the Schottky gun by a cold field emission gun (cold FEG) [89–92] has achieved a remarkable energy resolution of 9 meV as reported recently [31]. From an instrumental point of view, Cc corrector and cold FEG have similar advantages in increasing the beam coherency and provide premium imaging conditions for carbon-based nanomaterials or beam-sensitive materials in general.

**Figure 6:**
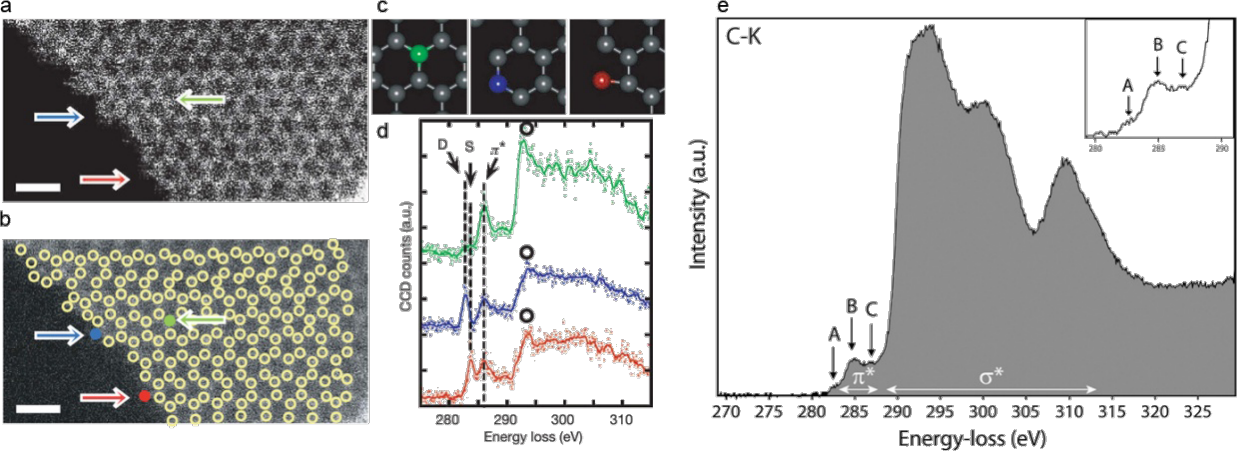
(a–d) Graphene edge spectroscopy; reproduced with permission from [29], Copyright (2010) Nature Publishing Group. (a) ADF-STEM image of a single graphene layer at the edge region. No image-processing has been done. Atomic positions are marked by circles in a smoothed image (b). Scale bars: 0.5 nm. (d) ELNES of carbon K1s spectra taken at the color-coded atoms indicated in (b). Green, blue and red spectra correspond to the normal sp^2^ carbon atom, a double-coordinated atom and a single-coordinated atom, respectively. These different states of atomic coordination are marked by colored arrows in (a) and (b) and illustrated in (c). (e) Experimental carbon K-edge ELNES acquired with monochromatic electron illumination. The σ* feature starting at 290 eV is typical for diamond. The π* contribution around 285 eV consists of 3 pre-peaks, at 282.7 eV (A), 285 eV (B), and 286.6 eV (C); reproduced with permission from [93], Copyright (2013) John Wiley and Sons.

Therefore, by combining the high energy resolution of EELS with the high spatial resolution of AC-(S)TEM, chemical information can be probed down to the limit. The remarkable potential of STEM–EELS to investigate carbon nanostructures was reported by Suenaga et al. These authors demonstrated atom-by-atom spectroscopy by probing a graphene edge [29]. Carbon rings were clearly imaged at a low voltage of 60 kV to minimize possible knock-on damage (Figure 6a,b). Fine-structure spectroscopic information of energy-loss near-edge structure (ELNES) spectra was collected simultaneously as probed by a highly converged electron probe. Carbon atoms with single-, double- and triple-coordination were distinguished through the information gathered on the electronic and bonding structures (Figure 6c,d). In comparison, ELNES performed on diamond is also shown here [93] as an example of carbon nanostructure with predominantly sp^3^ hybridization (Figure 6e). A unique σ* feature starting at 290 eV is typical for diamond, in contrast to that of graphene, which starts at 292 eV.

One step further, the doping of elements into carbon lattices can be investigated in great details using STEM–EELS, as demonstrated in the work of nitrogen-doped graphene [94] and nitrogen-doped CNT [95]. Substitutional nitrogen defects in graphene are identified by direct imaging using STEM, whereas the EELS spectrum collected at the neighboring carbon columns suggests a C–N bond [94]. Together with the help of first principles calculations, STEM–EELS further reveals the configurations of single N-substitutions in SWCNT as graphitic and pyrrolic [95]. A more striking result is reported in Si-doped graphene [96], in which a sp^3^-like trivalent Si substitute and a more complicated hybridized tetravalent Si impurity are clearly distinguished by atomic-resolved STEM–EELS spectra. It demonstrates the capability of EELS to reveal rich chemical information at an atomic scale. Although the work was performed on graphene/CNTs, chemical information provided by EELS can be used for the study of interfaces in carbon-based nanohybrids. For example, in [97] an attempt is given to resolve the interface of CNT–TiO_2_ hybrids. Segregation or mixed metal–carbon phases at the interface of nanohybrids can be evaluated.

Additionally, the introduction of the monochromator and high-resolution electron-loss spectrometers has greatly transformed the field of plasmonics and dielectric property measurements using valence EELS (VEELS). Different from ELNES which deals with the core-loss spectrum, VEELS focuses on the low-loss part of the EELS spectrum in the range of 0–50 eV and therefore is able to probe the optical properties, e.g., to measure the local band gap through a monochromated STEM [98–99]. Before the introduction of a monochromator and/or cold FEG, the energy resolution was limited to approximately 1 eV, which hinders the low-loss part of the EELS spectrum to be interpreted due to a broad tail near zero-loss peak. With the help of a monochromator and/or a cold FEG plasmonic properties can be studied, for instance in graphene. The low-loss EELS spectrum of graphene is dominated by plasmon excitations consisting of two peaks at about 4.5 eV and about 15 eV, referred to as π and π+σ surface plasmons, respectively, as confirmed both theoretically and experimentally in free-standing monolayer graphene [100–101]. Zhou et al. have demonstrated the surface plasmon resonances in monolayer graphene down to the atomic scale [102]. It is further revealed that a single point defect, as imaged by STEM, can act as an atomic antenna in the frequency range of petahertz, and thus enhance the surface plasmon resonance locally. However, more recently, Nelson et al., after extracting the dielectric function from STEM–EELS spectra and comparing it with the calculated results [103], claimed that the commonly referred π and π+σ peaks are not surface plasmons but single-particle interband excitations. Nevertheless, VEELS on graphene has opened up a venue to both the fundamental study and further applications in optoelectronics, plasmonics and transformative optics using carbon-based nanostructures.

Although it has been convincingly shown that STEM–EELS is able to reach the ultimate goal of materials characterization, combining atomic spatial resolution and millielectronvolt resolution on the energy scale, there are still limits that need to be treated with caution. For instance, the delocalization of inelastic scattering is in many cases larger than the probe size, indicating an actual spatial resolution possibly larger than the nominal value [23]. This has to be taken into account when interpreting data. The limit is then set by the physics behind rather than by the instrument. Nevertheless, the delocalization can be used to carry out “remote” spectroscopy by positioning the electron beam outside the sample and virtually “probe” the material, as demonstrated on vibrational spectroscopy acquired from various materials, both hard and soft [31]. Radiation damage can therefore be avoided in beam-sensitive materials, including carbon-based nanomaterials.

#### 3.3 In situ TEM

In situ studies of “working” processes at the nanometer scale or even the atomic scale are increasingly attracting attention. The dynamics of materials responding to external stimuli adds new perspectives and a new dimension to the study of carbon-based nanomaterials.

**Figure 7:**
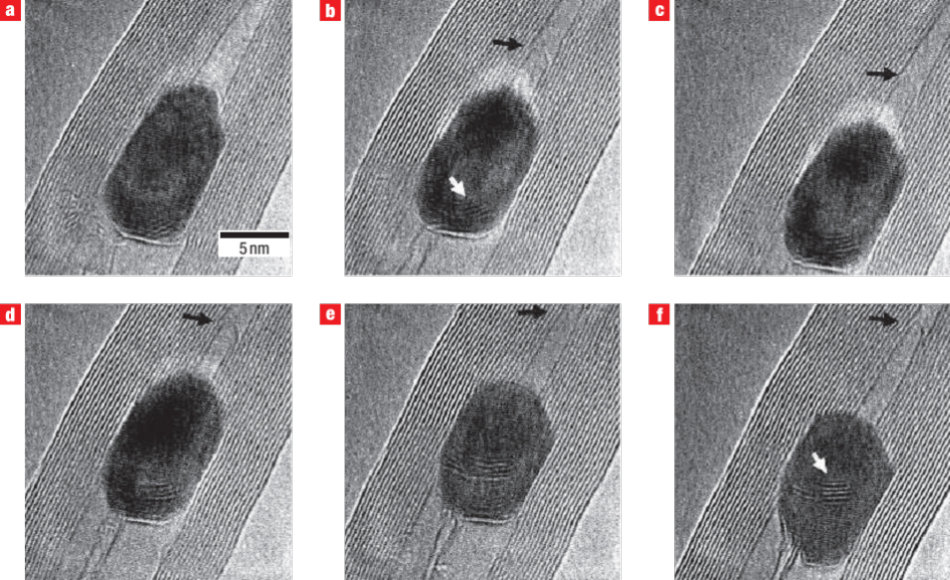
SWCNT growth from Fe with possible occurrence of a carbide phase. The HRTEM images show the growth of a SWCNT inside a MWCNT, which is partly filled with a Fe crystal under electron irradiation (ca. 200 A/cm^2^) at a specimen temperature of 600 °C. The tip of the growing SWCNT is indicated by a black arrow. Images taken (a) before the growth, (b) after 5 min, (c) after 6 min, (d) after 7 min, (e) after 13 min, (f) after 15 min of irradiation respectively; reproduced with permission from [105], Copyright (2007) Nature Publishing Group.

One of the most commonly applied stimuli is the temperature. Increasing the temperature has promoted self-healing of the knock-on damage in CNTs and related carbon-based nanohybrids in agreement with the thermodynamics of annealing. In situ heating to 600 °C allows CNTs to be studied at a high voltage of 300 kV while the structure remains defect-free [104]. Furthermore, carbon-based nanohybrids, particularly graphene or CNTs functionalized with metal nanoparticles, can be considered as a unique nanoreactor at elevated temperature. The interaction between energetic electrons and matter plays a unique role in triggering the reaction. Taking CNT growth as an example, the formation mechanism of CNTs was under debate for years until 2007, when Rodriguez-Manzo et al. monitored the nucleation and growth of a SWCNT through an in situ heating experiment using HRTEM [105]. As shown in Figure 7, a MWCNT filled with a Fe nanoparticle was regarded as a nanoreactor, where irradiation by the electron beam upon the MWCNT shells injects carbon atoms into the body of the nanoparticle. Diffusion of carbon atoms at high temperature subsequently leads to the formation of SWCNTs or MWCNTs at the tip of the nanoparticle inside the host nanotube. The experiment was performed in an entirely condensed phase process and revealed the growth mechanism of bulk diffusion of carbon through the body of catalytic particles, including Fe, Co and Ni.

Other external stimuli being introduced into in situ TEM, include mechanical stress [106–107], electrical stimuli [108], and chemical reactions in the gas phase or in liquid cells [50]. Although only a few studies using in situ TEM on carbon-based nanomaterials have been reported, an increase can certainly be expected. For instance, the evolution of defects (such as the shear strain present in CNTs as discussed in Section 3.1) along with the elastic/plastic deformation in CNT-reinforced composites under load can be well studied using similar techniques [109].

In contrast to the external stimuli, which are introduced through dedicated designs of the TEM specimen holder (thermal, mechanical, electrical) or specimen chamber (gas phase, environmental TEM), the contribution of electrons into the dynamic process, which can be regarded as the “internal stimulus”, is often overlooked. The interaction between electrons and carbon atoms may alter carbon-based nanostructures during imaging as discussed in Section 2. Unwanted destruction of the nanostructures can be avoided by imaging below the knock-on threshold of 80 kV, whereas active sites such as defects and functional species can still interact with electrons at lower voltages [43,110]. The advantage of this process, however, is the creation of a unique in situ platform in which active nanostructures can be studied at atomic resolution along the process [111]. It has found useful applications in the study of catalysis where functionalized carbon nanostructures are frequently employed as hosts for various catalysts [34].

**Figure 8:**
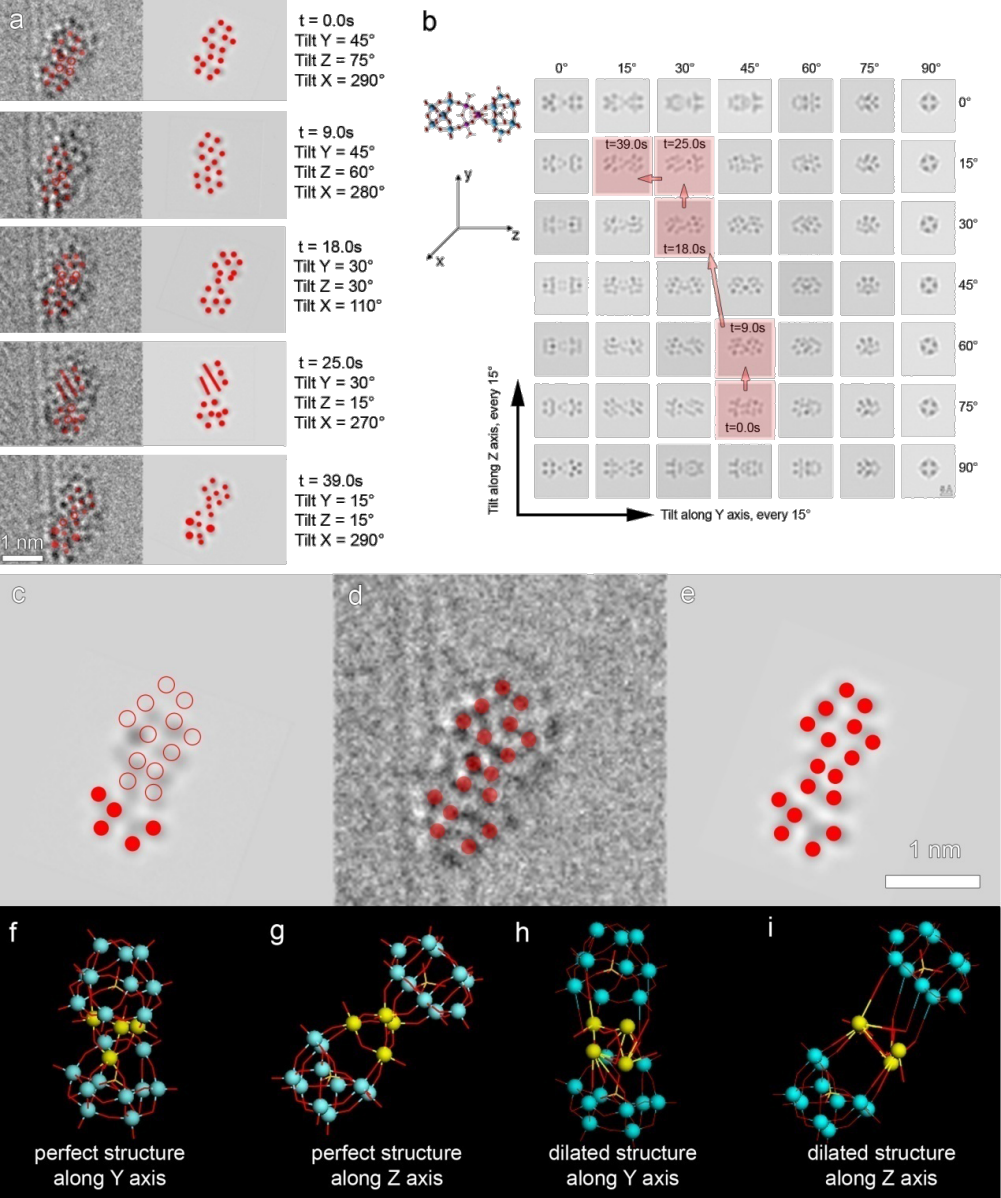
Dynamic study of Ru_4_POM functionalized on graphene. (a) Projections from a time sequence of functionalized Ru_4_POM together with their corresponding orientations determined from (b). (b) A plot summarizing simulated projections of an ideal Ru_4_POM molecule when tilted around the X and Y axes. The orientation changes from (a) are matched to the simulated patterns and highlighted by the red squares. (c) Simulated pattern of a Ru_4_POM whose orientation is found to fit into the HRTEM image shown in (a). The solid dot represents the fitted position of W, whereas the circle reveals a mismatch of the simulation and acquired pattern. (d) The acquired HRTEM image of Ru_4_POM to be fitted into the simulation shown in (c), where the position of W and Ru scheme proposed in (c) with a distorted Ru_4_ core. The fitted dots of Ru and W are indicated by solid red dots. (e) A simulated pattern of the dilated Ru_4_POM based on the scheme proposed in (c) with a distorted Ru_4_ core. The fitted dots of Ru and W are indicated by solid red dots. (f) The scheme of a perfect Ru_4_POM with viewing direction along Y axis and (g) along Z axis. (h) The scheme of the dilated Ru_4_POM based on the perfect structure shown in (f) and (g) with a manually distorted Ru_4_ core viewed along Y axis (h) and Z axis (i); reproduced with permission from [112], Copyright (2013) John Wiley and Sons.

We can demonstrate this using the example of functionalized graphene anchored by a water-splitting catalyst based on polyoxometalates (POMs). By imaging the nanohybrids at 80 kV, the supporting graphene is protected to a large degree and remains stable, whereas functionalized sites are more active when exposed to the electron beam. Sloan et al. for the first time reported the dynamical movement of discrete *C*_2_*_ν_* [γ-SiW_10_O_36_]^8−^ lacunary Keggin ions (a type of POM) at atomic resolution on a monolayer graphene oxide support [111]. A sequence of images recorded during the exposure to electrons demonstrate rotating, flipping and oscillation of the Keggin ions. Ke et al. reported an extensive study from 2D to 3D demonstrating the dynamics of Ru_4_POM functionalized on graphene, using the symmetry of Ru_4_POM as prior knowledge [112]. Ru_4_POM is composed of two rigid Keggion ions ([γ-SiW_10_O_36_]^8−^) interconnected by a tetraruthenium core. As shown in Figure 8a, the dynamic movements of an individual Ru_4_POM are captured over time. By comparing its projections to its simulated patterns at different tilted positions in 3D space (Figure 8b), the 3D configurations are reconstructed at each time point (highlighted by red squares). The dynamic behavior of each molecule in 3D can therefore be retrieved as a result of its interaction with the electron beam (indicated by arrows in Figure 8b).

The active sites of graphene are less resistant to the exposure of electrons, and therefore provide the anchoring points with a certain degree of flexibility allowing for the movement of the Ru_4_POM molecules. Additionally, a continuous deformation of the molecular structure is noticed throughout the imaging. A typical example is demonstrated in Figure 8c–i. The acquired projection of a Ru_4_POM (Figure 8d) is more elongated compared to the simulated pattern (Figure 8c). Detailed analysis at the atomic scale indicates that the deformation is mostly focused on the tetraruthenium core, which is the catalytic core as well. As shown in Figure 8f–i, a dilation is applied to the structure which distorts the Ru_4_ core, and provides a simulated pattern (Figure 8e) that fits well to the TEM image as shown in Figure 8d. It is remarkable that the Ru_4_POM molecules remain intact after intensive exposure to accelerated electrons, confirming the robustness of this catalyst and hints to the fact that the self-accommodation of the Ru_4_ core in its nanostructure may be responsible for the stability.

In situ TEM studies of carbon-based nanostructures have attracted substantial attention since the interaction of electrons and carbon lattices can be monitored at an atomic scale using AC-TEM. Electrons play the roles of probing tool and imaging tool simultaneously, which is unique for carbon-based nanomaterials. The introduction of this internal stimulus together with external stimuli has therefore allowed for the investigation of fundamental physics and chemistry at an atomic scale.

## Conclusion

In this review, the possibilities of modern electron microscopy for carbon-based nanomaterials have been discussed. AC-TEM has revolutionized our understanding of the materials by providing unprecedented spatial resolution and energy resolution at lower operating voltages. By minimizing the knock-on damage, extensive studies on the carbon-based nanomaterials at atomic scale are possible both structurally and chemically, from 2D to 3D. Further introduction of external stimuli has added multiple dimensions to the research field, where the dynamics of its response to stimuli can be revealed in detail. The interaction between electrons and carbon is essential when coming into the interpretation of any data, which makes the internal stimulus of electron beams a unique tool in both imaging and probing, allowing a study of fundamental physics and chemistry at atomic scale for carbon-based nanomaterials.
